# An Ad Hoc Random Initialization Deep Neural Network Architecture for Discriminating Malignant Breast Cancer Lesions in Mammographic Images

**DOI:** 10.1155/2019/5982834

**Published:** 2019-05-22

**Authors:** Andrea Duggento, Marco Aiello, Carlo Cavaliere, Giuseppe L. Cascella, Davide Cascella, Giovanni Conte, Maria Guerrisi, Nicola Toschi

**Affiliations:** ^1^Department of Biomedicine and Prevention, University of Rome Tor Vergata, Rome, Italy; ^2^IRCCS SDN, Naples, Italy; ^3^Idea 75 s.r.l., Bari, Italy; ^4^DEI-Politecnico di Bari, BARI, Italy; ^5^GEM ICT s.r.l., Bari, Italy; ^6^Department of Radiology, “Athinoula A. Martinos” Center for Biomedical Imaging, Boston, MA, USA; ^7^Harvard Medical School, Boston, MA, USA

## Abstract

Breast cancer is one of the most common cancers in women, with more than 1,300,000 cases and 450,000 deaths each year worldwide. In this context, recent studies showed that early breast cancer detection, along with suitable treatment, could significantly reduce breast cancer death rates in the long term. X-ray mammography is still the instrument of choice in breast cancer screening. In this context, the false-positive and false-negative rates commonly achieved by radiologists are extremely arduous to estimate and control although some authors have estimated figures of up to 20% of total diagnoses or more. The introduction of novel artificial intelligence (AI) technologies applied to the diagnosis and, possibly, prognosis of breast cancer could revolutionize the current status of the management of the breast cancer patient by assisting the radiologist in clinical image interpretation. Lately, a breakthrough in the AI field has been brought about by the introduction of deep learning techniques in general and of convolutional neural networks in particular. Such techniques require no a priori feature space definition from the operator and are able to achieve classification performances which can even surpass human experts. In this paper, we design and validate an ad hoc CNN architecture specialized in breast lesion classification from imaging data only. We explore a total of 260 model architectures in a train-validation-test split in order to propose a model selection criterion which can pose the emphasis on reducing false negatives while still retaining acceptable accuracy. We achieve an area under the receiver operatic characteristics curve of 0.785 (accuracy 71.19%) on the test set, demonstrating how an ad hoc random initialization architecture can and should be fine tuned to a specific problem, especially in biomedical applications.

## 1. Introduction

Breast cancer is one of the most common cancers in women, with more than 1,300,000 cases and 450,000 deaths each year worldwide [1]. In the era of precision medicine [2], the identification and stratification of breast lesions in the early stage of cancer development is an essential starting point for increasing the probability of therapeutic success. In this context, early detection of breast lesions through mammography has been seen to be associated with an extremely high probability of cure, with a 97% survival in five years [3]. To date, however, identification of breast cancer lesions is affected by an unsatisfactory rate of false-positive results.

Currently, X-ray mammography represents the standard breast screening technique. The false-positive and false-negative rates resulting by mammography are relatively high, especially for patients with very dense breasts [4, 5]. The sensitivity of mammography is further influenced by age and ethnicity of patients, personal history, implementation and (especially) expertise, and experience of the radiologist performing the exam. In addition, the mammographic exam does not provide any indication about probable disease evolution and/or outcome (and neither does it provide clues about possibly appropriate therapeutic choices). In this context, it is not surprising that the rate of false-negative or -positive results for mammography described in the literature is extremely variable. While it is evident that possibly high rates of false-negative results are critical, false positives also carry significant consequences. A recent retrospective investigation of registry data concerning 405,191 women aged 40 to 89 years, screened with digital mammography between 2003 and 2011, reported a rate of 12.12% of false-positive results. However, others studies indicate a rate of false positive of up to 20% in specific centers [6]. While a single study computed a very low rate of false-negative results (0.1 to 0.5%) regardless of the patient's age, several retrospective analyses indicated that mammographic examinations are associated with a high false-negative rate (between 8 and 16%), which is often quoted as an average 15%. These results, apparently controversial, can be explained by the numerous factors that influence the interpretation of mammographic images such as quality of instrumentation, radiologist's experience, and the availability of a second opinion [7–11]. Also, false-positive mammograms are often associated with increased short-term anxiety but no long-term anxiety and no measurable health utility decrement [11]. In a recent study, a false-positive result increased women's motivation to undergo future breast cancer screening, whilst it did not increase their self-reported motivation to travel to avoid a false-positive mammogram [12]. Also, in presence of false-positive cases, patients are frequently subjected to repeated invasive (bioptic examination) and/or stringent follow-up programs, such as additional mammography exams mammography or equivalent medical procedures which, on top of possibly generating health detriment on their own, also carry significant financial burden. The direct breast-care costs in the year following a false-positive screening mammogram are approximately 500$ higher than in the case of a true-negative result [13].

In view of the above, the introduction of novel artificial intelligence (AI) technologies applied to the diagnosis and possibly prognosis of breast cancer could revolutionize the current status of the management of the breast cancer patient. The support of AI in the diagnostic path of breast cancer patients can potentially both reduce the healthcare costs due to misdiagnosis and promote the achievement of new precision medicine protocols [14]. In this context, the disruptive innovation in computer vision brought about through what is known as deep learning [15–17], and in particular, a class of methods known as deep convolutional neural networks (CNNs) [18] is very quickly making its way into the world of medical imaging. Accordingly, in a preliminary study, Chougrad et al. [13] described a CAD based on deep CNN able to discriminate between malignant and benignant breast mass in mammographic images with high accuracy. Likewise, other papers employed massive transfer learning approaches (GoogleNet and AlexNet) [19–21] and compared them to in-house, random initialization models showing that the latter achieves fairly poor performance. Other authors focused on a relatively small dataset and an “in-house” architecture measuring the relationship between network depth and model performance [22]. Still, published results are often hard to validate and replicate also due to the lack of a shared, standard curated dataset of informative mammographic images, and transfer learning approaches may not perform equally well when applied to datasets which are too distant in nature from the application at hand.

The main aim of this study was to design an ad hoc random initialization “in-house” deep neural network architecture to classify/detect breast lesion and explore whether satisfactory performance can be obtained without having to include the inaccurately trained, albeit powerful, public models currently available for transfer learning. Given the strong dependence of CNN performance on the specific task, we aimed to distill what are the key characteristics of a CNN suitable for breast lesion classification. We based our investigation on the recently released Curated Breast Imaging Subset of the Digital Database for Screening Mammography, which is curated by trained radiologists as well as pathologists.

## 2. Methods

### 2.1. Dataset

The training and testing of our CNN is done over the Curated Breast Imaging Subset of DDSM Digital Database for Screening Mammography (CBIS-DDSM) [23, 24], which is a collection of mammograms from several sources (Massachusetts General Hospital, Wake Forest University School of Medicine, Sacred Heart Hospital, and Washington University of St. Louis School of Medicine). The database collects both mediolateral oblique (MLO) and craniocaudal (CC) views of each breast. Each breast view is annotated with regions of interest (ROIs) for masses manually drawn (freehand) by expert radiologists and automatically included in a rectangular section of the image. Other annotations include the Breast Imaging Reporting and Data System (BI-RADS) descriptors for mass shape, mass margin, and breast density; overall BI-RADS assessment ranged from 0 to 5; rating of the subtlety of the abnormalities ranged from 1 to 5.  Table 1 provides summary of the annotations available for each image.

### 2.2. Workflow and Architecture Overview

**Figure 1:**
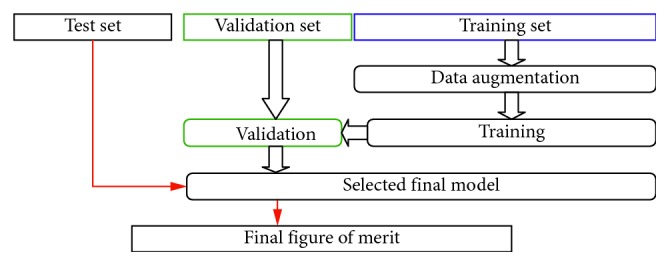
Workflow of our method. The original training set provided by CBIS-DDSM is further divided into a new “training set” and a “validation set.” The new training set is employed to fit the model parameters, and the validation set is employed to validate and compare the performance of each model on an unbiased set of images. The final model is chosen accordingly to its performance of the validation set and its performance quantified in an unbiased manner on the test set. Overall, the split was as follows: training set (1158 images), validation set (160 images), and test set (378 images).

Our model was developed by combining the TensorFlow [25] and Keras [26] libraries; the whole workflow (Figure 1) consists of the following: (i) image preprocessing as described above; (ii) data augmentation; (iii) CNN training; (iv) performance evaluation with respect to a validation set, which allows to compare models trained on the training set; and (v) final evaluation of the best model on the test set. The CNN training is further composed of several steps (which also depends on the specific CNN architecture which can be grouped in (1) convolutional layers and (2) neural layers). Each step is described in the following paragraphs.

#### 2.2.1. Image Preprocessing

**Figure 2:**
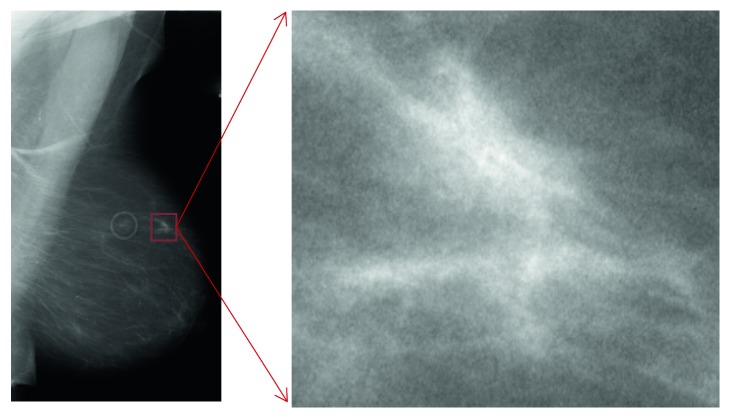
Example whole raw images and ROI extraction to be passed to image augmentations.

Every mass/ROI (Figure 2) is labeled either as “benign” or “malignant” according to pathological findings. As input, we employed all the presegmented ROIs containing images of masses, retaining only the “benign”-“malignant” label and hence stripping any other information (Figure 1). Starting from a training set of 1318 images and a test set of 378 images, we created a training set of 1158 images, a validation set of 160 images, and retained the original test set of 378 images.

#### 2.2.2. Data Augmentation

**Figure 3:**
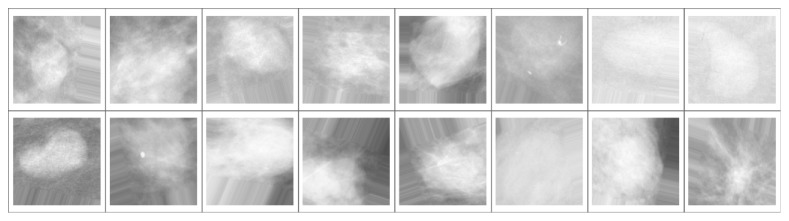
Example of a batch of 16 images from the training set. The ROI from which each image has been generated has been randomly rescaled (independently over the two axes), rotated by a random angle, randomly flipped, and resampled to fit into a pixel frame with aspect ratio 1. Any remaining area not filled by the image is padded with an array of pixels drawn from the edge of the image.

It is common practice to synthetically increase the information available to the CNN by applying multiple transformations to the training set [27]. This practice is called “augmentation” and serves the purpose of providing the learning algorithm with as many informative images as possible in order to prevent overfitting (i.e., an excessive specialization of the CNN to the data at hand, which occurs when the training dataset is not sufficiently large to allow for generalization). Accordingly, for each extracted ROI, we perform data augmentation by transforming the training images employing random rotations, rescalings, and shear deformations (it is important to note that since CNNs are not invariant for affine transformation, this process is actually able to inject new training information into the dataset).  Figure 3 shows an example of a batch of images resulting from the augmentation process.

#### 2.2.3. Training

The process of training consists in tuning the weights of the model (see following paragraphs), to maximize the loss function of the model and hence the accuracy of the automatic classification/diagnosis formulated by the model. Batches of images from the CNN training set are fed into the algorithm, and the weights of the model are found by a trial and error in the attempt to improve its accuracy. Each “attempt” is commonly called “epoch”. After each epoch, the weights of the model are updated.

**Figure 4:**
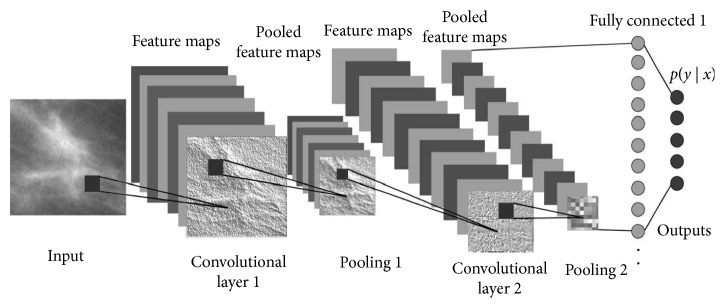
Overall architecture of the model (adapted from [28]).

*(1) Convolutional Layers*. Convolutional layers are the first stages of the actual image processing pipeline (Figure 4), and their role is to distill information regarding spatially correlated features of the input image. Convolutional layers function in a way that resembles the physiology of early pathways of the visual cortical areas in humans, where neurons respond to simple tuning—e.g., a neuron might be sensitive to vertical contrasts while another to horizontal contrasts. For example, convolution processes may highlight edges, or smooth the image, or make contrasts in a specific direction more prominent. At each layer, convolved images are subsampled to reduce resolution and passed to the next layer. Each convolutional layer extracts features using as input a linear combination of the outputs of the previous layer. Recursively, more and more (but smaller and smaller) images are produced, each containing information about an intricate combination of features. To the human eye, the images produced after the last layers typically look completely unrelated to the original input. A more technical description of this process can found in [29]. The convolutional part of the CNN is described by the number of convolutional layers, the number of convolutional kernels in each layer and their sizes, the details of the activation functions, and other image processing steps (e.g., how the subsampling is done and whether there is a global-normalization step).

*(2) Neural Layers*. The output of the last convolutional layer is the input to a series of one or few layers of neuronal arrays. A neuronal array is a set of weighted switch-like discriminators that, much like to the firing of a neuron excited by a suprathreshold stimulus, activate when a certain combination of features is active. Again, stacking two or more neuronal layers allows to extract more and more sophisticated combinations of features. Such neural layers are called “fully-connected” because each neuron is linked *a priori* with any element (a voxel in an image or a neuron) of the previous layer. The weights of those links are tuned during the training process. In our model, the very last layer is composed by a single neuron with a sigmoid activation, i.e., its output is a number between 0 and 1, which describes the algorithm's educated guess regarding the malignancy of (the mass depicted in) the image (0: completely benign, 1: completely malignant). Varying the threshold on this continuous sigmoid function allows the construction of receiver operating characteristic (ROC) curves.

#### 2.2.4. Performance Evaluation during Model Training and Model Selection

At each epoch, we test the diagnostic accuracy of the model on a separate validation set (see above) which, importantly, is not used (i.e., it is completely “unseen”) for training, thus providing an unbiased evaluation tool. For example, a high accuracy on the training set coupled with a low accuracy on the validation set is a good indication of overfitting has occurred.

It is important to note that, for real-life problems, there is no simple way to choose the best model architecture. Very similar architectures can perform differently, while very different architectures in terms of depth, number of layers, or number of parameters perform could perform almost equally. In this paper, we heuristically explored the space of number of possible architectures and trained them in order to gain insights into what an optimal CNN architecture for classification of breast lesions may be. In particular, we explored (though not exhaustively) the space of the following parameters: number of convolutional layers (2–5), size of the input image (from 78 to 612 pixels, depending on architecture and dimensions of images after the last convolutional layer, which in turn ranged from 1 to 8 pixels), number of convolutional kernels per each layer (from 4 to 64, not necessary identical on every layer), size of the convolutional kernel (from 3 to 11, not necessary identical on every layer), size of pooling (from 2 to 4, depending on the image size and kernel size), method for the last layer vectorization (global mean, global max, or flattening), number of fully connected layers before the last single-neuron layer (from 1 to 3), and numbers of neurons in each fully connected layer (from 200 to 5, typically decreasing with depth of the layer), for a total of 260 tested architectures. Every architecture was evaluated according to its performance on the validation set according to two separate criteria: (a) highest area under the ROC curve (AUC) (“model 1”) and (b) best *F*2 score amongst all best *F*2 statistics attained by every single architecture (“model 2”). The *F*2 score is defined as *F*2=5*∗* precision *∗* recall/4*∗*precision+recall. Within each model, the optimal operating point was chosen according to the *F*1 score (i.e., maximizing the harmonic average of precision and sensitivity, a commonly adopted criterion which compromises between sensitivity and the ability to discriminate a true positive result) for model 1 and *F*2 score for model 2.

## 3. Results

**Figure 5:**
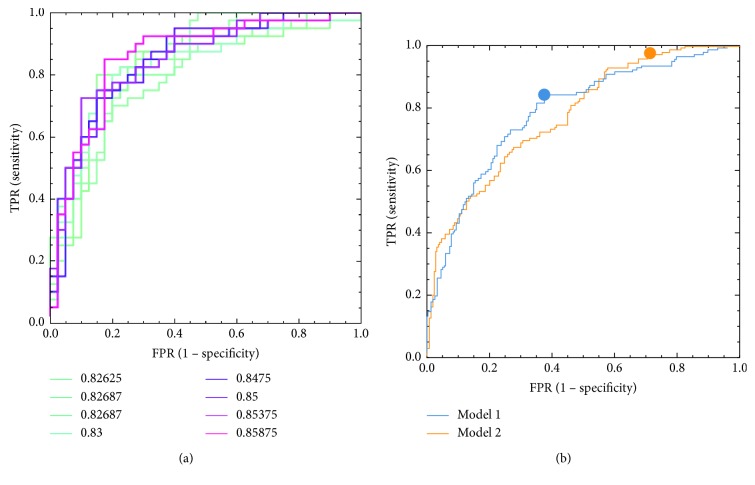
(a) Receiver operating characteristic (ROC) curves for a subsample of the architectures tested on the validation set (AUCs obtained on the validation set are shown in the legend). (b) ROC curve related to our best performing model (model 1: selected according to AUC on the validation set and model 2: selected according to *F*2 statistics on the validation set) when evaluated on the test set.

**Figure 6:**
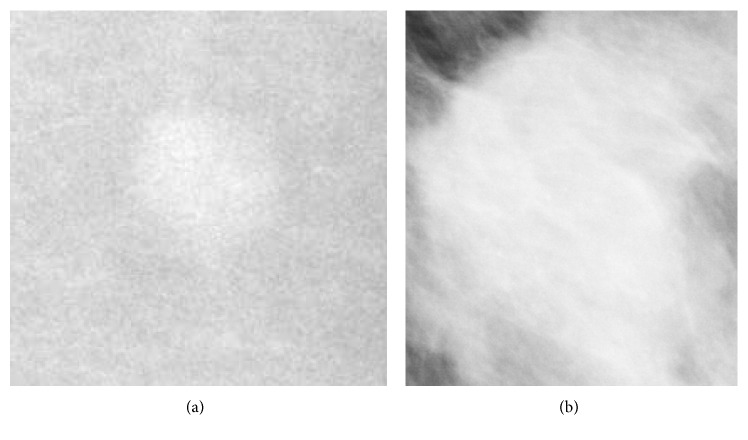
Example images that are easy to classify: (a) image of a benign lesion that is easily categorized as a benign lesion (score 2.2 × 10^−9^ from model 1 on a scale from 0 to 1); (b) image of a malignant lesion that is easily categorized as a malignant lesion (score 1.0 from model 1 on a scale from 0 to 1).

**Figure 7:**
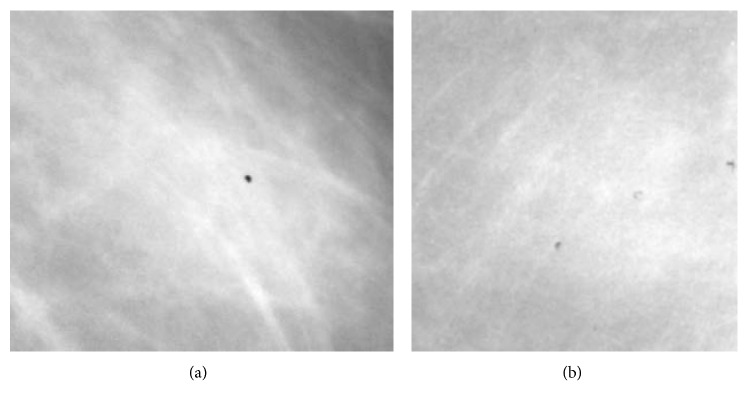
Example images that are very difficult to classify: (a) image of a benign lesion that is falsely categorized as a malignant lesion (score 0.99992 from model 1 on a scale from 0 to 1); (b) image of a malignant lesion that is falsely categorized as a benign lesion (score .0133 from model 1 on a scale from 0 to 1).

Both “model 1” and “model 2” happened to share the same convolutional architecture: 3 convolutional layers with 64 kernels each; size of kernels in each layer was 7 × 7, 5 × 5, and 3 × 3, respectively; the parameter dropout factor on each convolution was 25%; after rectified linear unit (ReLU) activation, on each layer, a max pooling method with size 4 × 4, 3 × 3, and 2 × 2 (and same stride) was employed. “Model 1” and “model 2” differed only in terms of the size of the input images and of the neuronal architecture: “model 1” had an input image of 238 × 238 pixels and fully connected neuronal layers composed by 50 and 10 neurons each before the last single-neuron layer. “Model 2” had an input image of 286 × 286 pixels and fully connected neuronal layers composed by 50 and 20 neurons each before the last single-neuron layer. Training the models took approximately 78 hours (4000 training epochs) on a 40-CPU dedicated HP bladesystem. Examples of our result on the validation set as well as final performance of our best models on the test set are shown in Figure 5. Examples of images which are “easy” to classify correctly are shown in Figure 6. Examples of images which are “difficult” to classify correctly are shown in Figure 7.

Our final “model 1” achieved an AUC of 0.785. Detailed performance statistics for this model when selecting an optimal operating point according to the best *F*1 score method are presented in Table 2. Our final “model 2” achieved an AUC curve of 0.774. Detailed performance statistics for this model when selecting an optimal operating point according to the best *F*2 score (which is a weighted average between sensitivity—which is emphasized 4-fold—and positive predictive value (PPV)) method are also presented in Table 2.

## 4. Discussion

While the classical machine learning (ML) paradigm is based on providing a result (i.e., a classification) given a human-defined set of features extracted from input data, CNNs are able to capture intricate relations between image features that are typically invisible to the human eye. Moreover, CNN architectures need not to be problem specific. However, their adaptability with respect to the image classification tasks, and their complete independence from the burden as well as possible bias of human-defined features, comes with the cost of a vast number of parameters which, in turns, require a large amount of training data. Given a certain CNN architecture, if the demand of training data is not met, the performance of the algorithm in terms of classification accuracy might plunge to chance levels. In this pilot study, we have explored the possibility of designing ad hoc CNN architecture with random initialization while studying heuristically which characteristics, out of the multitude of CNN varieties, may be important for breast lesion classification and may warrant further investigation. We employed rigorous validation and test set splits and achieved an area under the ROC curve of 0.78. Additionally, the optimal cutoff point as calculated with an *F*1 statistics was associated with 62.44% specificity and 84.4% sensitivity. Given the health as well as psychological implications of a false-negative diagnosis in breast cancer (see Introduction), we also strived to select a model which could pose more emphasis on avoided false negatives while still being selected rigorously. We therefore evaluated our model performance at an operating point determined by maximizing the *F*2 statistic, obtaining a sensitivity of 99.7%. While the specificity of this model may seem low, it is important to note that, when performing model as well as operating point (i.e., cutoff) selection, it is critical to keep the end-user's needs and priorities in mind. We therefore put forward that, in a condition like breast cancer where a false negative may have devastating consequences which are overall much more burdensome than those of a false positive, a criterion like the *F*2 statistic (or similar) may be the instrument of choice.

As noted in the introduction, a few papers based almost exclusively on transfer learning have obtained comparable or higher performance on breast cancer classification as compared to our results. While transfer learning can provide steeper learning rates and asymptotically higher performance when approaching a new classification task and a small training set, it is likely that a dedicated learning framework would reach asymptotically higher performance when a large enough training set is made available. Further, one might speculate that the type of background knowledge and the realm of the application are also influential: for a lesion detection problem in mammograms, an architecture well-trained to distinguish (say) cars from the pedestrian might make a worse transfer learning source than, for example, an equally well-trained architecture to distinguish benign from malignant lung nodules.

Of note, the capabilities of a CNN in particular, and of deep learning in general, can, e.g., also be extended to predict molecular alterations (e.g., genetic changes) as long as the training data has been annotated both clinically and genomically in an accurate manner [30]. This could greatly enhance the management of breast cancer patients, in which the choice of therapeutic strategy is currently based on molecular characteristics of breast tumors, which in turn established by histological analysis of biopsies or surgical samples. Specifically, immunohistochemical reactions allow to evaluate the expression of targets for biological (cerB2), antihormonal (estrogen receptor), or radiochemical therapies (Ki67) [31–33]. Therefore, one can envisage an algorithm able to predict the molecular features of breast cancer tissues by the analysis of digital mammographic images, which could be conceivably realized by training a CNN jointly with histopathological and molecular data. The introduction of this type of diagnostic approaches has the potential to introduce radical changes in the organization of imaging diagnostic, anatomic pathology, as well as oncology departments. Specifically, the possibility to provide oncologists with possible molecular profiles and/or treatment options at the time of mammography could significantly reduce the need for bioptic investigation, hence optimizing the overall resources available to the healthcare facility. Most importantly, such CAD frameworks could ameliorate the patient's quality of life by reducing both the number of invasive procedures such as (often repeated) biopsies as well as the average wait before therapy inception. Also, deep learning has the potential to seamlessly integrate data from multimodal imaging of breast cancer, such as mammography and molecular imaging (PET, CT, and MR), with digitalized histological images. The algorithms could be trained to emphasize and highlight morphological signs whose identification is commonly time-consuming to the naked eye but may result in diagnostically actionable items (e.g., microvessel density, neoangiogenesis, lymphovascular invasion, chromatin alteration, or mitotic figures). This type of workflow would not only render pathology and imaging work quick and more accurate but also redefine the role of pathologists to experts able to agglomerate and interpret genetic/molecular, morphological, and imaging information to produce a more integrated and accurate diagnosis [34, 35].

In summary, our pilot study can lay the foundation for the development of new multimodal and multidisciplinary diagnostic tools able to move yet another step towards the goal of realizing a true personalized medicine approach able to take into account the unique peculiarities of every human being.

## Figures and Tables

**Table 1:** Summary of the annotations available for each image in the CBIS-DDSM dataset. As all these annotations are derived from the image, none of these features were imputed into our classifier.

Patient_id	Anonymous alphanumeric code
Breast_density	4 (153), 2 (757), 3 (449), 1 (337)
Left or right breast	Left (817), right (879)
Image view	CC(784), MLO(912)
Abnormality id	1 (1570), 2 (84), 4 (10), 3 (28), 5 (2), 6 (2) (integer index used to label multiple lesions within the same image)
Abnormality type	Mass (1696)
Mass shape	Irregular (526), round (169), lobulated (399), oval (423), architectural_distortion(158), asymmetric_breast_tissue(26), lymph_node(45)
Mass margins	Focal_asymmetric_density (27), n/a (4), spiculated (407), circumscribed (455), ill_defined (472), obscured (308), microlobulated (143), n/a (60)
Assessment	5 (374), 4 (702), 0 (162), 3 (364), 2 (91), 1 (3)
Pathology	Malignant (784), benign (771), benign_without_callback (141)
Subtlety	5 (687), 4 (453), 2 (141), 3 (358), 1 (55), 0 (2)

**Table 2:** Performance statistics for our best performing models as evaluated on the test set.

*Model 1 (best AUC overall on tde validation set, point witd bestF1 score on tde test set)*									
Accuracy	PPV (precision)	FDR	TPR (recall, sensitivity)	FNR (missrate)	FPR (fall out)	TN (specificity)	*F*1 score	*F*2 score	*F*5 score
71.19%	59.80%	40.20%	84.40%	15.60%	37.56%	62.44%	70.00%	77.98%	63.50%

*Model 2 (best F2 score overall on the validation set, point with best F2 score on the test set)*									
Accuracy	PPV (precision)	FDR	TPR (recall, sensitivity)	FNR (missrate)	FPR (fallout)	TN (specificity)	*F*1 score	*F*2 score	*F*5 score
55.93%	47.40%	52.60%	97.16%	2.84%	71.36%	28.64%	63.72%	80.30%	52.81%

## Data Availability

The images from the Curated Breast Imaging Subset data used for training the algorithms are from previously reported studies and datasets, which have been cited. The processed data are described in (DOI: 10.1038/sdata.2017.177) and currently available at https://wiki.cancerimagingarchive.net/display/Public/CBIS-DDSM.
